# 3,3′-[(4-Nitro­phen­yl)methyl­ene]bis­(4-hy­droxy-2*H*-chromen-2-one)

**DOI:** 10.1107/S1600536811054778

**Published:** 2012-01-07

**Authors:** N. Ravikumar, G. Gopikrishna, K. Anand Solomon

**Affiliations:** a Sankar Foundation Research Institute, Naiduthota, Vepagunta, Visakhapatnam, Andhra Pradesh 530 047, India

## Abstract

The molecular conformation of the title compound, C_25_H_15_NO_8_, is stabilized by strong intramolecular O—H⋯O hydrogen bonds, resulting in the formation of *S*_1_^1^(7) ring motifs. In the crystal, π–π stacking inter­actions are observed between adjacent nitrobenzene and pyranone rings with a centroid–centroid distance of 3.513 (12) Å. The dihedral angles between the nitrobenzene ring and the coumarin ring systems are 65.61 (8) and 66.11 (8)° while the coumarin ring systems are inclined at 65.69 (8)°.

## Related literature

For the synthesis of benzyl­idene-bis-(4-hy­droxy­coumarin) derivatives, see: Mehrabi & Abusaidi (2010 ▶); Završnik *et al.* (2011 ▶). For hydrogen bonds, see: Desiraju & Steiner (1999 ▶). For graph-set analysis of hydrogen bonds, see: Etter *et al.* (1990 ▶); Bernstein *et al.* (1995 ▶). For the biological activity of substituted benzyl­idene-bis-(4-hy­droxy­coumarin) derivatives, see: Borges *et al.* (2005 ▶); Nolan *et al.* (2009 ▶); Prakash *et al.* (2008 ▶); Zhao *et al.* (1997 ▶).
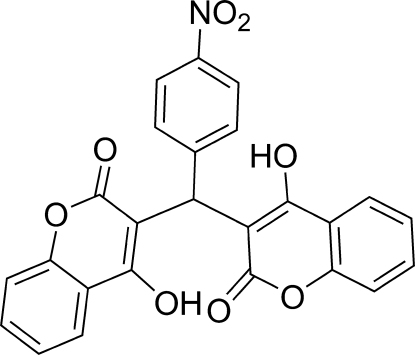


## Experimental

### 

#### Crystal data

C_25_H_15_NO_8_
*M* *_r_* = 457.38
Orthorhombic, 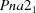

*a* = 14.0061 (6) Å
*b* = 14.1511 (6) Å
*c* = 10.4179 (4) Å
*V* = 2064.85 (15) Å^3^
*Z* = 4
Mo *K*α radiation
μ = 0.11 mm^−1^
*T* = 295 K
0.35 × 0.30 × 0.25 mm

#### Data collection

Bruker Kappa APEXII CCD diffractometer
Absorption correction: multi-scan (*SADABS*; Bruker, 2004 ▶) *T*_min_ = 0.902, *T*_max_ = 0.973
15733 measured reflections
3316 independent reflections
2913 reflections with *I* > 2σ(*I*)
*R*_int_ = 0.030

#### Refinement

*R*[*F* ^2^ > 2σ(*F* ^2^)] = 0.033
*wR*(*F* ^2^) = 0.089
*S* = 1.04
3316 reflections
310 parameters
1 restraint
H-atom parameters constrained
Δρ_max_ = 0.22 e Å^−3^
Δρ_min_ = −0.14 e Å^−3^

### 

Data collection: *APEX2* (Bruker, 2004 ▶); cell refinement: *SAINT* (Bruker, 2004 ▶); data reduction: *SAINT*; program(s) used to solve structure: *SIR92* (Altomare *et al.*, 1993 ▶); program(s) used to refine structure: *SHELXL97* (Sheldrick, 2008 ▶); molecular graphics: *ORTEP-3* (Farrugia, 1997 ▶) and *Mercury* (Macrae *et al.*, 2006 ▶); software used to prepare material for publication: *PLATON* (Spek, 2009 ▶).

## Supplementary Material

Crystal structure: contains datablock(s) I, global. DOI: 10.1107/S1600536811054778/rk2323sup1.cif

Structure factors: contains datablock(s) I. DOI: 10.1107/S1600536811054778/rk2323Isup2.hkl

Supplementary material file. DOI: 10.1107/S1600536811054778/rk2323Isup3.cml

Additional supplementary materials:  crystallographic information; 3D view; checkCIF report

## Figures and Tables

**Table 1:** Hydrogen-bond geometry (Å, °)

*D*—H⋯*A*	*D*—H	H⋯*A*	*D*⋯*A*	*D*—H⋯*A*
O3—H3*A*⋯O5	0.82	1.79	2.597 (2)	166
O6—H6*A*⋯O1	0.82	1.80	2.617 (2)	173

**Fig. 1.**
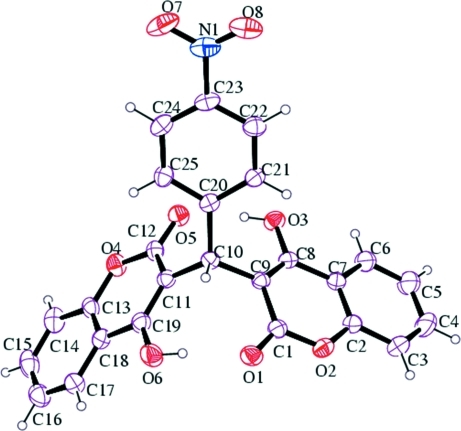
Molecular structure of title compound with the atom numbering scheme. Displacement ellipsoids are drawn at 30% probability level. H atoms are presented as a small spheres of arbitrary radius.

**Fig. 2.**
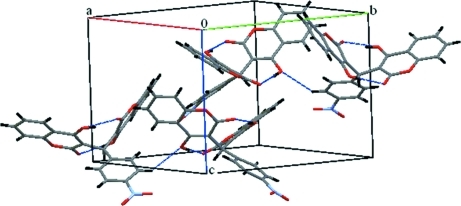
Crystal packing diagram of the title compound.

## supplementary crystallographic information

### Comment

Sevaral methods were reported in the literature (Mehrabi *et al.* 2010) and (Završnik *et al.* 2011) for the synthesis of the title compound. Coumarin ring forms an important pharmacophore in several naturally occurring as well as synthetic molecules (Prakash *et al.* 2008). These coumarin derivaties showed numerous therapeutic applications such as anticoagulant and antibacterial agents (Borges *et al.* 2005). Several multifunctionalized coumarin derivatives were reported to exhibit anti-*HIV* properties (Zhao *et al.* 1997) and also as inhibitors of quinone oxidoreductase-1 (Nolan *et al.* 2009).

In title compound, C_25_H_15_NO_8_, I, two 4-hydroxycoumarin moieties are linked through a methylene bridge on which one hydrogen atom has been replaced with a phenyl ring bearing *p*-nitro group (Fig. 1). The 4-hydroxycoumarin moieties are stabilized by intramolecular hydrogen bonding by forming S^1^_1_(7) ring motifs (Etter *et al.* 1990) and (Bernstein *et al.* 1995) between hydroxyl and carbonyl oxygen atoms. The crystal structure of I is stabilized by C–H···O and π–π interactions (Fig. 2). The range of H···O distances (Table 1) found in I agrees with those found for C–H···O hydrogen bonds (Desiraju & Steiner, 1999). The supramolecular chains were extended by π–π-interactions, where the distance between the two centroids namely (C1/O2/C2/C7-C9) and (C20-C25) of the two corresponding coplanar rings is 3.513 (12)Å.

### Experimental

The 4-hydroxycoumarin (2 m.mol, 0.324 g) and 4-nitrobenzaldehyde (1 mmol, 0.151 g) were refluxed in ethanol (5 ml) at 333 K for 12 h. After completion of the reaction as monitored by *TLC*, the reaction mixture was cooled to room temperature. The obtained precipitate was collected by suction filtration and dried. The pure product was obtained by recrystallization from dichloromethane in 92% yield.

### Refinement

All H atoms were positioned geometrically. H atoms attached to C atoms were placed in calculated positions with C–H = 0.93Å (aromatic) and C–H = 0.98Å (methine) with *U*_iso_(H) = 1.2*U*eq(C) and allowed to ride. The O–H distances were restrained to 0.82Å and refined as riding atoms with *U*_iso_(H) = 1.5*U*eq(O) in the final cycles of refinement. The 1539 Friedel pairs were merged during structure refinement.

### Crystal data

**Table:**

C_25_H_15_NO_8_	*F*(000) = 944
*M**_r_* = 457.38	*D*_x_ = 1.471 Mg m^−^^3^
Orthorhombic, *P**n**a*2_1_	Mo *K*α radiation, λ = 0.71073 Å
Hall symbol: P 2c -2n	Cell parameters from 6438 reflections
*a* = 14.0061 (6) Å	θ = 2.1–24.2°
*b* = 14.1511 (6) Å	µ = 0.11 mm^−^^1^
*c* = 10.4179 (4) Å	*T* = 295 K
*V* = 2064.85 (15) Å^3^	Block, orange
*Z* = 4	0.35 × 0.30 × 0.25 mm

### Data collection

**Table:**

Bruker Kappa APEXII CCD diffractometer	3316 independent reflections
Radiation source: fine-focus sealed tube	2913 reflections with *I* > 2σ(*I*)
graphite	*R*_int_ = 0.030
ω and φ scans	θ_max_ = 24.3°, θ_min_ = 2.1°
Absorption correction: multi-scan (*SADABS*; Bruker, 2004)	*h* = −15→16
*T*_min_ = 0.902, *T*_max_ = 0.973	*k* = −16→16
15733 measured reflections	*l* = −12→12

### Refinement

**Table:**

Refinement on *F*^2^	Secondary atom site location: difference Fourier map
Least-squares matrix: full	Hydrogen site location: inferred from neighbouring sites
*R*[*F*^2^ > 2σ(*F*^2^)] = 0.033	H-atom parameters constrained
*wR*(*F*^2^) = 0.089	*w* = 1/[σ^2^(*F*_o_^2^) + (0.0543*P*)^2^ + 0.1473*P*] where *P* = (*F*_o_^2^ + 2*F*_c_^2^)/3
*S* = 1.04	(Δ/σ)_max_ < 0.001
3316 reflections	Δρ_max_ = 0.22 e Å^−^^3^
310 parameters	Δρ_min_ = −0.14 e Å^−^^3^
1 restraint	Extinction correction: *SHELXL97* (Sheldrick, 2008), Fc^*^=kFc[1+0.001xFc^2^λ^3^/sin(2θ)]^-1/4^
Primary atom site location: structure-invariant direct methods	Extinction coefficient: 0.0066 (11)

### Special details

**Table:**

Geometry. All s.u.'s (except the s.u. in the dihedral angle between two l.s. planes) are estimated using the full covariance matrix. The cell s.u.'s are taken into account individually in the estimation of s.u.'s in distances, angles and torsion angles; correlations between s.u.'s in cell parameters are only used when they are defined by crystal symmetry. An approximate (isotropic) treatment of cell s.u.'s is used for estimating s.u.'s involving l.s. planes.
Refinement. Refinement of *F*^2^ against ALL reflections. The weighted *R*-factor *wR* and goodness of fit *S* are based on *F*^2^, conventional *R*-factors *R* are based on *F*, with *F* set to zero for negative *F*^2^. The threshold expression of *F*^2^ > σ(*F*^2^) is used only for calculating *R*-factors(gt) *etc*. and is not relevant to the choice of reflections for refinement. *R*-factors based on *F*^2^ are statistically about twice as large as those based on *F*, and *R*-factors based on ALL data will be even larger.

### Fractional atomic coordinates and isotropic or equivalent isotropic displacement parameters (Å^2^)

**Table:**

	*x*	*y*	*z*	*U*_iso_*/*U*_eq_	
C1	0.65097 (16)	0.52018 (16)	0.9180 (2)	0.0425 (5)	
C2	0.67257 (16)	0.67214 (16)	0.8278 (2)	0.0465 (5)	
C3	0.73555 (19)	0.7470 (2)	0.8087 (3)	0.0672 (7)	
H3	0.7965	0.7461	0.8442	0.081*	
C4	0.7053 (2)	0.8221 (2)	0.7359 (4)	0.0817 (9)	
H4	0.7464	0.8724	0.7210	0.098*	
C5	0.6141 (2)	0.82367 (19)	0.6844 (3)	0.0757 (9)	
H5	0.5947	0.8751	0.6353	0.091*	
C6	0.55268 (19)	0.75111 (17)	0.7049 (3)	0.0585 (6)	
H6	0.4915	0.7531	0.6704	0.070*	
C7	0.58132 (16)	0.67400 (16)	0.7773 (2)	0.0455 (5)	
C8	0.52015 (15)	0.59312 (15)	0.80351 (19)	0.0412 (5)	
C9	0.55171 (14)	0.52169 (14)	0.87827 (19)	0.0374 (5)	
C10	0.49380 (14)	0.43608 (14)	0.9198 (2)	0.0377 (5)	
H10	0.5301	0.4097	0.9918	0.045*	
C11	0.49566 (14)	0.35862 (15)	0.81912 (19)	0.0403 (5)	
C12	0.42673 (16)	0.36412 (15)	0.7157 (2)	0.0422 (5)	
C13	0.49157 (18)	0.22343 (14)	0.6267 (2)	0.0492 (6)	
C14	0.4862 (2)	0.15815 (18)	0.5260 (3)	0.0643 (7)	
H14	0.4379	0.1614	0.4650	0.077*	
C15	0.5557 (2)	0.08853 (19)	0.5208 (3)	0.0742 (9)	
H15	0.5537	0.0436	0.4556	0.089*	
C16	0.6277 (2)	0.08488 (19)	0.6106 (3)	0.0698 (8)	
H16	0.6742	0.0382	0.6047	0.084*	
C17	0.6315 (2)	0.14788 (16)	0.7066 (3)	0.0623 (7)	
H17	0.6806	0.1441	0.7665	0.075*	
C18	0.56350 (17)	0.21869 (15)	0.7178 (2)	0.0486 (6)	
C19	0.56297 (16)	0.29003 (15)	0.8164 (2)	0.0484 (6)	
C20	0.39601 (14)	0.45796 (15)	0.9770 (2)	0.0389 (5)	
C21	0.37848 (15)	0.54447 (15)	1.0351 (2)	0.0453 (5)	
H21	0.4233	0.5926	1.0277	0.054*	
C22	0.29604 (17)	0.56044 (17)	1.1036 (2)	0.0516 (6)	
H22	0.2853	0.6184	1.1431	0.062*	
C23	0.22999 (15)	0.48919 (18)	1.1123 (2)	0.0503 (6)	
C24	0.24222 (17)	0.40444 (18)	1.0508 (2)	0.0534 (6)	
H24	0.1951	0.3582	1.0537	0.064*	
C25	0.32609 (17)	0.38952 (15)	0.9845 (2)	0.0472 (5)	
H25	0.3358	0.3318	0.9438	0.057*	
N1	0.14454 (16)	0.5031 (2)	1.1934 (2)	0.0652 (6)	
O1	0.69024 (11)	0.45293 (11)	0.96905 (17)	0.0531 (4)	
O2	0.70638 (10)	0.59589 (11)	0.89430 (15)	0.0509 (4)	
O3	0.43458 (11)	0.59699 (11)	0.74979 (15)	0.0502 (4)	
H3A	0.4116	0.5438	0.7469	0.075*	
O4	0.42395 (12)	0.29414 (11)	0.62710 (16)	0.0528 (4)	
O5	0.37047 (12)	0.42867 (11)	0.70029 (16)	0.0515 (4)	
O6	0.63447 (12)	0.28370 (12)	0.90099 (19)	0.0663 (5)	
H6A	0.6486	0.3367	0.9266	0.099*	
O7	0.08403 (15)	0.44080 (18)	1.1938 (3)	0.0931 (7)	
O8	0.13895 (14)	0.57467 (17)	1.2562 (2)	0.0814 (6)	

### Atomic displacement parameters (Å^2^)

**Table:**

	*U*^11^	*U*^22^	*U*^33^	*U*^12^	*U*^13^	*U*^23^
C1	0.0420 (12)	0.0473 (13)	0.0382 (11)	0.0001 (11)	0.0025 (9)	−0.0094 (10)
C2	0.0413 (12)	0.0472 (13)	0.0511 (13)	−0.0003 (11)	0.0030 (11)	−0.0054 (11)
C3	0.0438 (14)	0.0689 (17)	0.089 (2)	−0.0088 (13)	0.0035 (14)	−0.0068 (16)
C4	0.0630 (19)	0.0618 (17)	0.120 (3)	−0.0202 (14)	0.0069 (18)	0.0188 (19)
C5	0.0685 (19)	0.0583 (16)	0.100 (2)	−0.0043 (14)	0.0060 (17)	0.0207 (16)
C6	0.0534 (15)	0.0545 (14)	0.0677 (16)	0.0010 (12)	0.0029 (12)	0.0087 (13)
C7	0.0428 (12)	0.0449 (12)	0.0488 (12)	−0.0003 (10)	0.0089 (10)	−0.0043 (10)
C8	0.0337 (11)	0.0490 (13)	0.0409 (12)	0.0026 (10)	0.0026 (9)	−0.0021 (10)
C9	0.0342 (11)	0.0427 (11)	0.0354 (11)	0.0005 (9)	0.0016 (9)	−0.0049 (9)
C10	0.0369 (12)	0.0417 (11)	0.0345 (10)	0.0026 (9)	−0.0070 (9)	0.0004 (9)
C11	0.0389 (11)	0.0412 (12)	0.0409 (12)	−0.0014 (9)	0.0022 (10)	0.0040 (9)
C12	0.0509 (14)	0.0389 (11)	0.0368 (11)	−0.0038 (11)	0.0033 (10)	0.0010 (10)
C13	0.0604 (14)	0.0345 (11)	0.0526 (13)	−0.0015 (10)	0.0142 (12)	0.0055 (10)
C14	0.0851 (18)	0.0583 (15)	0.0494 (14)	−0.0100 (15)	0.0073 (14)	−0.0014 (13)
C15	0.112 (3)	0.0490 (15)	0.0616 (18)	−0.0094 (16)	0.0349 (18)	−0.0098 (13)
C16	0.0773 (19)	0.0537 (15)	0.079 (2)	0.0051 (14)	0.0275 (18)	0.0036 (15)
C17	0.0576 (15)	0.0484 (14)	0.0809 (18)	0.0018 (12)	0.0105 (13)	0.0003 (15)
C18	0.0493 (13)	0.0425 (12)	0.0541 (13)	−0.0059 (11)	0.0042 (11)	0.0039 (11)
C19	0.0480 (13)	0.0442 (12)	0.0531 (14)	−0.0025 (11)	−0.0030 (11)	0.0054 (11)
C20	0.0373 (11)	0.0486 (12)	0.0307 (10)	−0.0013 (10)	−0.0040 (9)	0.0031 (9)
C21	0.0401 (12)	0.0518 (13)	0.0441 (12)	−0.0054 (10)	0.0010 (10)	−0.0027 (11)
C22	0.0475 (13)	0.0621 (14)	0.0452 (13)	0.0028 (11)	0.0019 (11)	−0.0027 (12)
C23	0.0352 (12)	0.0741 (16)	0.0417 (11)	0.0025 (11)	0.0000 (10)	0.0101 (13)
C24	0.0461 (13)	0.0652 (16)	0.0488 (13)	−0.0126 (12)	0.0014 (11)	0.0093 (12)
C25	0.0521 (14)	0.0474 (12)	0.0421 (12)	−0.0071 (11)	0.0002 (11)	0.0001 (10)
N1	0.0462 (13)	0.0952 (18)	0.0543 (13)	0.0153 (13)	0.0055 (11)	0.0188 (14)
O1	0.0451 (9)	0.0569 (9)	0.0572 (9)	0.0081 (8)	−0.0130 (8)	−0.0037 (8)
O2	0.0381 (8)	0.0581 (9)	0.0565 (9)	−0.0053 (8)	−0.0031 (7)	−0.0013 (8)
O3	0.0418 (9)	0.0545 (9)	0.0544 (10)	0.0002 (7)	−0.0068 (7)	0.0087 (8)
O4	0.0626 (10)	0.0494 (9)	0.0464 (9)	0.0000 (8)	−0.0093 (8)	−0.0036 (8)
O5	0.0520 (10)	0.0515 (9)	0.0510 (9)	0.0035 (8)	−0.0109 (7)	0.0003 (8)
O6	0.0612 (11)	0.0539 (10)	0.0837 (13)	0.0115 (9)	−0.0241 (10)	−0.0028 (10)
O7	0.0484 (12)	0.1247 (18)	0.1063 (17)	−0.0157 (12)	0.0204 (12)	0.0188 (15)
O8	0.0626 (12)	0.1100 (17)	0.0715 (14)	0.0265 (12)	0.0137 (10)	−0.0006 (13)

### Geometric parameters (Å, °)

**Table:**

C1—O1	1.221 (3)	C13—C14	1.400 (3)
C1—O2	1.346 (3)	C14—C15	1.386 (4)
C1—C9	1.451 (3)	C14—H14	0.9300
C2—O2	1.367 (3)	C15—C16	1.376 (4)
C2—C7	1.382 (3)	C15—H15	0.9300
C2—C3	1.393 (3)	C16—C17	1.341 (4)
C3—C4	1.373 (4)	C16—H16	0.9300
C3—H3	0.9300	C17—C18	1.388 (3)
C4—C5	1.385 (4)	C17—H17	0.9300
C4—H4	0.9300	C18—C19	1.440 (3)
C5—C6	1.356 (4)	C19—O6	1.337 (3)
C5—H5	0.9300	C20—C25	1.379 (3)
C6—C7	1.386 (3)	C20—C21	1.388 (3)
C6—H6	0.9300	C21—C22	1.376 (3)
C7—C8	1.456 (3)	C21—H21	0.9300
C8—O3	1.324 (3)	C22—C23	1.371 (3)
C8—C9	1.350 (3)	C22—H22	0.9300
C9—C10	1.521 (3)	C23—C24	1.371 (4)
C10—C11	1.517 (3)	C23—N1	1.478 (3)
C10—C20	1.525 (3)	C24—C25	1.379 (3)
C10—H10	0.9800	C24—H24	0.9300
C11—C19	1.353 (3)	C25—H25	0.9300
C11—C12	1.449 (3)	N1—O8	1.209 (3)
C12—O5	1.217 (2)	N1—O7	1.223 (3)
C12—O4	1.355 (3)	O3—H3A	0.8200
C13—O4	1.378 (3)	O6—H6A	0.8200
C13—C18	1.386 (3)		
			
O1—C1—O2	116.14 (19)	C15—C14—H14	121.3
O1—C1—C9	124.6 (2)	C13—C14—H14	121.3
O2—C1—C9	119.3 (2)	C16—C15—C14	120.9 (3)
O2—C2—C7	121.88 (19)	C16—C15—H15	119.5
O2—C2—C3	117.0 (2)	C14—C15—H15	119.5
C7—C2—C3	121.1 (2)	C17—C16—C15	120.8 (3)
C4—C3—C2	118.2 (2)	C17—C16—H16	119.6
C4—C3—H3	120.9	C15—C16—H16	119.6
C2—C3—H3	120.9	C16—C17—C18	121.0 (3)
C3—C4—C5	120.8 (3)	C16—C17—H17	119.5
C3—C4—H4	119.6	C18—C17—H17	119.5
C5—C4—H4	119.6	C13—C18—C17	118.4 (2)
C6—C5—C4	120.8 (3)	C13—C18—C19	116.8 (2)
C6—C5—H5	119.6	C17—C18—C19	124.8 (2)
C4—C5—H5	119.6	O6—C19—C11	123.8 (2)
C5—C6—C7	119.9 (3)	O6—C19—C18	114.8 (2)
C5—C6—H6	120.1	C11—C19—C18	121.4 (2)
C7—C6—H6	120.1	C25—C20—C21	117.97 (19)
C2—C7—C6	119.3 (2)	C25—C20—C10	121.14 (19)
C2—C7—C8	117.3 (2)	C21—C20—C10	120.55 (18)
C6—C7—C8	123.4 (2)	C22—C21—C20	121.3 (2)
O3—C8—C9	124.8 (2)	C22—C21—H21	119.3
O3—C8—C7	114.90 (19)	C20—C21—H21	119.3
C9—C8—C7	120.27 (19)	C23—C22—C21	118.7 (2)
C8—C9—C1	119.29 (19)	C23—C22—H22	120.7
C8—C9—C10	125.86 (18)	C21—C22—H22	120.7
C1—C9—C10	114.72 (18)	C24—C23—C22	121.9 (2)
C11—C10—C9	111.69 (17)	C24—C23—N1	119.0 (2)
C11—C10—C20	115.59 (16)	C22—C23—N1	119.1 (2)
C9—C10—C20	115.37 (17)	C23—C24—C25	118.3 (2)
C11—C10—H10	104.2	C23—C24—H24	120.8
C9—C10—H10	104.2	C25—C24—H24	120.8
C20—C10—H10	104.2	C24—C25—C20	121.7 (2)
C19—C11—C12	119.1 (2)	C24—C25—H25	119.1
C19—C11—C10	123.00 (18)	C20—C25—H25	119.1
C12—C11—C10	117.63 (17)	O8—N1—O7	123.9 (2)
O5—C12—O4	116.10 (19)	O8—N1—C23	118.2 (2)
O5—C12—C11	124.8 (2)	O7—N1—C23	117.9 (3)
O4—C12—C11	119.12 (19)	C1—O2—C2	121.44 (17)
O4—C13—C18	122.2 (2)	C8—O3—H3A	109.5
O4—C13—C14	116.4 (2)	C12—O4—C13	120.87 (19)
C18—C13—C14	121.3 (2)	C19—O6—H6A	109.5
C15—C14—C13	117.5 (3)		
			
O2—C2—C3—C4	176.3 (2)	O4—C13—C18—C17	−176.8 (2)
C7—C2—C3—C4	−1.3 (4)	C14—C13—C18—C17	0.7 (3)
C2—C3—C4—C5	0.8 (5)	O4—C13—C18—C19	1.8 (3)
C3—C4—C5—C6	0.0 (5)	C14—C13—C18—C19	179.3 (2)
C4—C5—C6—C7	−0.4 (5)	C16—C17—C18—C13	−0.5 (4)
O2—C2—C7—C6	−176.6 (2)	C16—C17—C18—C19	−179.0 (2)
C3—C2—C7—C6	1.0 (4)	C12—C11—C19—O6	174.1 (2)
O2—C2—C7—C8	3.1 (3)	C10—C11—C19—O6	−0.3 (3)
C3—C2—C7—C8	−179.3 (2)	C12—C11—C19—C18	−4.3 (3)
C5—C6—C7—C2	−0.1 (4)	C10—C11—C19—C18	−178.79 (19)
C5—C6—C7—C8	−179.8 (2)	C13—C18—C19—O6	−179.0 (2)
C2—C7—C8—O3	−177.99 (19)	C17—C18—C19—O6	−0.5 (3)
C6—C7—C8—O3	1.7 (3)	C13—C18—C19—C11	−0.4 (3)
C2—C7—C8—C9	2.4 (3)	C17—C18—C19—C11	178.1 (2)
C6—C7—C8—C9	−177.9 (2)	C11—C10—C20—C25	28.4 (3)
O3—C8—C9—C1	172.24 (19)	C9—C10—C20—C25	161.27 (19)
C7—C8—C9—C1	−8.2 (3)	C11—C10—C20—C21	−158.45 (19)
O3—C8—C9—C10	−3.4 (3)	C9—C10—C20—C21	−25.6 (3)
C7—C8—C9—C10	176.20 (19)	C25—C20—C21—C22	3.1 (3)
O1—C1—C9—C8	−168.8 (2)	C10—C20—C21—C22	−170.3 (2)
O2—C1—C9—C8	8.8 (3)	C20—C21—C22—C23	−0.8 (3)
O1—C1—C9—C10	7.3 (3)	C21—C22—C23—C24	−2.7 (3)
O2—C1—C9—C10	−175.05 (17)	C21—C22—C23—N1	175.6 (2)
C8—C9—C10—C11	84.8 (2)	C22—C23—C24—C25	3.7 (3)
C1—C9—C10—C11	−91.0 (2)	N1—C23—C24—C25	−174.5 (2)
C8—C9—C10—C20	−49.9 (3)	C23—C24—C25—C20	−1.3 (3)
C1—C9—C10—C20	134.28 (18)	C21—C20—C25—C24	−2.0 (3)
C9—C10—C11—C19	89.4 (2)	C10—C20—C25—C24	171.3 (2)
C20—C10—C11—C19	−136.0 (2)	C24—C23—N1—O8	173.4 (2)
C9—C10—C11—C12	−85.1 (2)	C22—C23—N1—O8	−4.9 (3)
C20—C10—C11—C12	49.5 (3)	C24—C23—N1—O7	−5.9 (3)
C19—C11—C12—O5	−171.1 (2)	C22—C23—N1—O7	175.9 (2)
C10—C11—C12—O5	3.7 (3)	O1—C1—O2—C2	174.34 (19)
C19—C11—C12—O4	7.8 (3)	C9—C1—O2—C2	−3.5 (3)
C10—C11—C12—O4	−177.41 (17)	C7—C2—O2—C1	−2.5 (3)
O4—C13—C14—C15	177.5 (2)	C3—C2—O2—C1	179.8 (2)
C18—C13—C14—C15	−0.1 (4)	O5—C12—O4—C13	172.42 (19)
C13—C14—C15—C16	−0.8 (4)	C11—C12—O4—C13	−6.6 (3)
C14—C15—C16—C17	1.0 (4)	C18—C13—O4—C12	1.8 (3)
C15—C16—C17—C18	−0.3 (4)	C14—C13—O4—C12	−175.8 (2)

### Hydrogen-bond geometry (Å, °)

**Table:**

*D*—H···*A*	*D*—H	H···*A*	*D*···*A*	*D*—H···*A*
O3—H3A···O5	0.82	1.79	2.597 (2)	166
O6—H6A···O1	0.82	1.80	2.617 (2)	173
C10—H10···O1	0.98	2.34	2.809 (3)	109
C10—H10···O6	0.98	2.49	2.928 (3)	107

## References

[bb1] Altomare, A., Cascarano, G., Giacovazzo, C. & Guagliardi, A. (1993). *J. Appl. Cryst.* **26**, 343–350.

[bb2] Bernstein, J., Davis, R. E., Shimoni, L. & Chang, N.-L. (1995). *Angew. Chem. Int. Ed. Engl.* **34**, 1555–1573.

[bb3] Borges, F., Roleira, F., Milhazes, N., Santana, L. & Uriarte, E. (2005). *Curr. Med. Chem.* **12**, 887–916. 10.2174/0929867053507315 15853704

[bb4] Bruker (2004). *APEX2*, *SAINT* and *SADABS* Bruker AXS Inc., Madison, Wisconsin, USA.

[bb5] Desiraju, G. A. & Steiner, T. (1999). *The Weak Hydrogen Bond in Structural Chemistry and Biology* New York: Oxford University Press Inc

[bb6] Etter, M. C., MacDonald, J. C. & Bernstein, J. (1990). *Acta Cryst.* B**46**, 256–262. 10.1107/s0108768189012929 2344397

[bb7] Farrugia, L. J. (1997). *J. Appl. Cryst.* **30**, 565.

[bb9] Macrae, C. F., Edgington, P. R., McCabe, P., Pidcock, E., Shields, G. P., Taylor, R., Towler, M. & van de Streek, J. (2006). *J. Appl. Cryst.* **39**, 453–457.

[bb10] Mehrabi, H. & Abusaidi, H. (2010). *J. Iran. Chem. Soc.* **4**, 890–894.

[bb11] Nolan, A. K., Doncaster, R. J., Dunstan, S. M., Scot, A. K., Frenkel, D., Siegel, D., Ross, D., Barnes, J., Levy, C. & Leys, D. (2009). *J. Med. Chem.* **57**, 7142–7156. 10.1021/jm9011609 19877692

[bb12] Prakash, O., Kumar, R. & Prakash, V. (2008). *Eur. J. Med. Chem.* **43**, 435–440. 10.1016/j.ejmech.2007.04.004 17555846

[bb13] Sheldrick, G. M. (2008). *Acta Cryst.* A**64**, 112–122. 10.1107/S0108767307043930 18156677

[bb14] Spek, A. L. (2009). *Acta Cryst.* D**65**, 148–155. 10.1107/S090744490804362X PMC2631630 19171970

[bb15] Završnik, D., Muratović, S., Damjan Makuc, D., Plavec, J., Cetina, M., Nagl, A., Clercq, E. D., Balzarini, J. & Mintas, M. (2011). *Molecules*, **16**, 6023–6040. 10.3390/molecules16076023 PMC6264767 21772234

[bb16] Zhao, H., Neamati, N., Hong, H., Mazumder, A., Wang, S., Sunder, S., Milne George, W. A., Pommier, Y. & Burke, T. R. Jr (1997). *J. Med. Chem.* **40**, 242–249. 10.1021/jm960450v 9003523

